# A simple and fast spectroscopy-based technique for Covid-19 diagnosis

**DOI:** 10.1038/s41598-021-95568-5

**Published:** 2021-08-18

**Authors:** Driss Lahlou Kitane, Salma Loukman, Nabila Marchoudi, Alvaro Fernandez-Galiana, Fatima Zahra El Ansari, Farah Jouali, Jamal Badir, Jean-Luc Gala, Dimitris Bertsimas, Nawfal Azami, Omar Lakbita, Omar Moudam, Rachid Benhida, Jamal Fekkak

**Affiliations:** 1 Operations Research Center, MIT, Muckley Bldg, 1 Amherst St, Cambridge, MA 02142 USA; 2 Anoual Laboratory, Boulevard d’Alexandrie, 20360 Casablanca, Morocco; 3 LIGO Laboratory, MIT, 185 Albany Street, Cambridge, MA 02139 USA; 4 Centre for Applied Molecular Technologies (CTMA), Université Catholique de Louvain, Louvain, Belgium; 5 Photonics Labs, INPT, Madinat Al Irfane, Rabat, Morocco; 6 Chemical and Biochemical Sciences Department (CBS), Mohammed VI Polytechnic University, UM6P. Lot 660, Hay Moulay Rachid, 43150 Benguerir, Morocco; 7 Nice Institute of Chemistry, University Côte d’Azur, Nice, France

**Keywords:** Biological techniques, Biophysics, Biotechnology, Medical research

## Abstract

The coronavirus pandemic, which appeared in Wuhan, China, in December 2019, rapidly spread all over the world in only a few weeks. Faster testing techniques requiring less resources are key in managing the pandemic, either to enable larger scale testing or even just provide developing countries with limited resources, particularly in Africa, means to perform tests to manage the crisis. Here, we report an unprecedented, rapid, reagent-free and easy-to-use screening spectroscopic method for the detection of SARS-CoV-2 on RNA extracts. This method, validated on clinical samples collected from 280 patients with quantitative predictive scores on both positive and negative samples, is based on a multivariate analysis of FTIR spectra of RNA extracts. This technique, in agreement with RT-PCR, achieves 97.8% accuracy, 97% sensitivity and 98.3% specificity while reducing the testing time post RNA extraction from hours to minutes. Furthermore, this technique can be used in several laboratories with limited resources.

## Introduction

According to the World Health Organization (WHO), a pandemic is the worldwide spread of a new disease, characterized by a rapid propagation and high mortality rate. Transmitted by viruses, bacteria and other pathogens, it kills millions of people. Several pandemics are well-known in human history, from various plagues in the Middle Ages to the Spanish influenza pandemic in the last century, with 50 million deaths^1^ ascribed to H1N1 type virus^2^. It is worth noting that RNA viruses are particularly dangerous since they have high mutation rates, enhanced virulence and evolvability. They have been involved in most severe epidemics such as HIV^3^, HCV^4^, Ebola^5^, H1N^6,7^, etc. Most of these diseases resulted from an animal-to-human transmission, and a lack of accessible and rapid diagnostic tests generally hampered adequate health response and efficient management of the disease.

Presently, the world is experiencing an unprecedented health crisis, SARS-CoV-2 (severe acute respiratory syndrome coronavirus-2) referred to as a Covid-19 disease^8–10^. The first cases were reported in December 2019 in Wuhan, China; then it rapidly spread worldwide in only a few days. The fast spread of Covid-19 is mainly attributed to the mode of transmission of the virus and the high volume of business and tourism airline traffic. Moreover, emerging mutations known as Covid-19 variants (United Kingdom, Brazil and South Africa) increased transmissibility of the virus and improve its ability to escape the host immune system. The number of infected people is still increasing, with more than 103 million confirmed cases and more than 2.26 million confirmed deaths worldwide^11^. Beside the global health crisis, this pandemic has also triggered devastating social and economic impacts across the globe.

Even with significant medical resources in the developed world, most sophisticated healthcare systems are being overwhelmed by the magnitude of the pandemic. From limited healthcare workers to the lack of medical capacity, many developing countries are facing unprecedented health challenges in managing the Covid-19 situation^12^. The first confirmed Covid-19 cases in Africa were reported in Egypt (Feb 14, 2020), mostly imported from Europe^13^. Between February and March 2020, the pandemic spread rapidly, and most of the African countries reported several confirmed Covid-19 cases, with an increased rate of infections and an offset, time-shifted spread when compared to European neighbors confirming the origin of the pandemic^13^.

Currently, a new chapter in Covid-19 fight is open with the vaccine phase^14–16^. The COVID-19 vaccine race is launched in early 2021 and several countries worldwide are already undergoing massive vaccination programs. Indeed, since the identification of SARS-CoV-2 several vaccines have started to be rolled out. Some of them were made available in only few months for emergency reasons instead of years (> 10 years) and others are under clinical trials^14–17^. Even if the vaccines are expected to slow down human-to-human transmission, the rapid increase of virus mutations is still challenging for the efficacy of the vaccines and the overall management of the pandemic^18^. Moreover, little is known about the safety, immunity, protection and transmission level of vaccinated patients^19,20^. Therefore, rapid testing is still a critical cornerstone in the overall management of this pandemic, enabling healthcare to trace and contain the virus as well as to efficiently prepare the current vaccination phase.

Several diagnostic assays have been reported for SARS-Cov-2 detection^21^. Among them, virus isolation^22^, RNA quantification^23^, antigen detection and serological methods for detecting IgM and IgG are the most widely used methods in laboratory diagnosis and in virologic studies^24^. Very recently, new innovative technologies have been reported, with reliable efficiency in decreasing the overall time of the analysis and in pushing the limits of detection. Some of them are RT-PCR-like such as nano-PCR^25^, multiplex RT-PCR^26^, isothermal amplification^27^; and others rely on the integration of interesting CRISPR-based detection^28–30^.

Currently, diagnosis of SARS-Cov-2 virus is mainly based on the quantitative polymerase chain reaction (RT-PCR) for the detection of viral nucleic acids^31,32^. These methods have several limitations such as sample handling, requiring samples in the acute phase, and testing time, which ranges from 2 to 4 h for a simple PCR acquisition to more than 12 h for the overall processing and handling time. Moreover, this technique also requires the use of expensive kits that are mostly sourced from western and Chinese suppliers and cannot be guaranteed for African countries particularly during lockdown periods. In other words, the diagnosis of SARS-Cov-2 infected patients in African countries using the PCR technique is inadequate and requires, in several countries, more than 5 days to get the test results to the patients.

**Figure 1:**
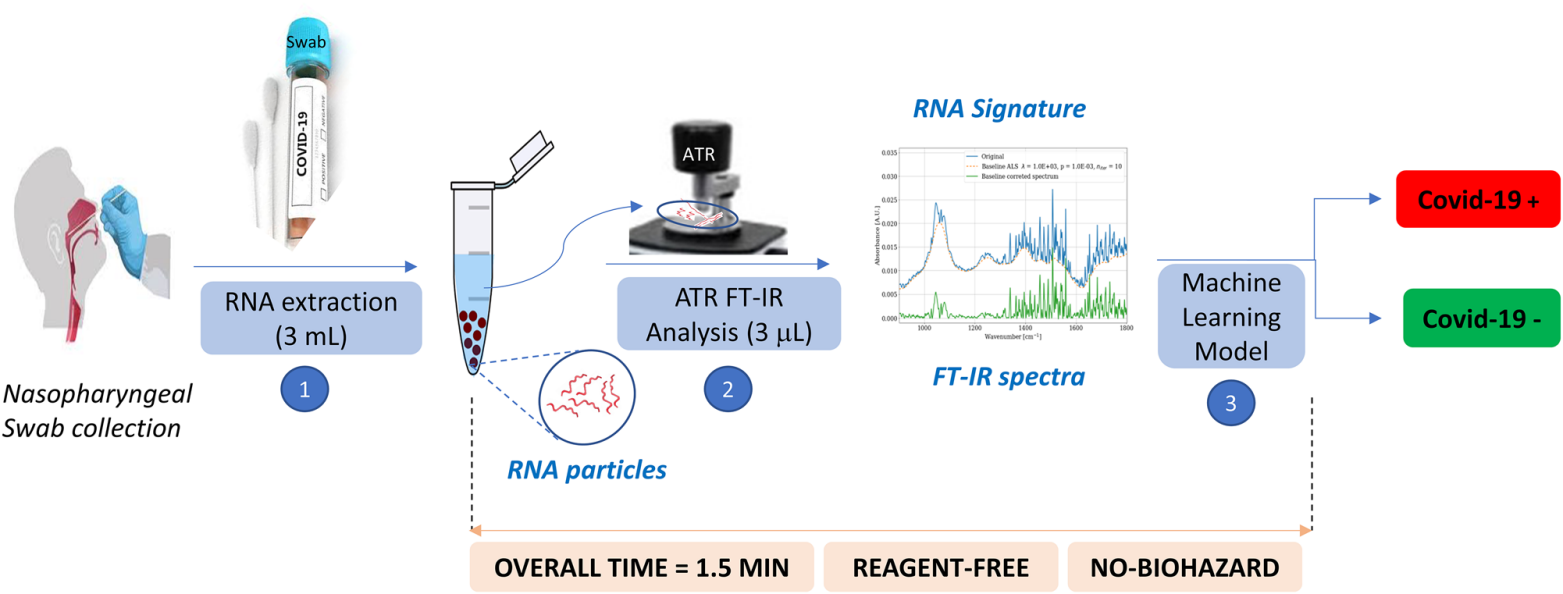
Sequential FT-IR-based assay for detection of SARS-CoV-2: sample collection, RNA extraction, FTIR analysis and then machine learning.

In line with these considerations, herein we report an unprecedented and highly accessible screening method for the detection of SARS-CoV-2 using extracted RNA samples. This method is based on a straightforward combination of infrared (IR) spectroscopy and machine learning (ML). Compared to RT-PCR, this method is faster (1.5 min vs 2–4 h post-RNA extraction), requires no reagents, and less biohazard waste is generated at the end of the test (Fig. 1).

The use of IR spectroscopy for viral detection is relatively new^33–35^. Although the method has several advantages, it might require a sizeable number of samples to enhance its sensitivity, specificity and accuracy. In this work, we use, for the first time, machine learning to build classification models able to predict the patient's infection, based on the IR spectra of the extracted RNAs. To the best of our knowledge, this technology, based on a dual and complementary combination of FT-IR and machine learning on RNA samples, has not been reported yet, given the high diagnosis performance results achieved, suggests spectroscopy as a promising tool for viral diagnosis. We also used sparse classification techniques to improve the interpretability of models. To the best of our knowledge, this also the first time such technique is used on FTIR-based diagnosis techniques. We perform experiments on two sets of samples. A first set of 280 clinical samples is used to assess the sensitivity, specificity and accuracy. A second set of synthetic RNA samples is used to evaluate the limit of detection and further assess the selectivity against 15 other respiratory viruses.

## Results

### Clinical data

Research on spectroscopy based viral detection relies on different fields including microbiology, spectroscopy, data processing and machine learning. The efficiency of this method relies on the quality of the clinical specimens, the protocol used, hardware configuration, signal pre-processing techniques and the choice and tuning of statistical algorithms. We outline here the combination that achieves the highest performance. Other set-ups are detailed and discussed in Supplementary Information.

**Figure 2:**
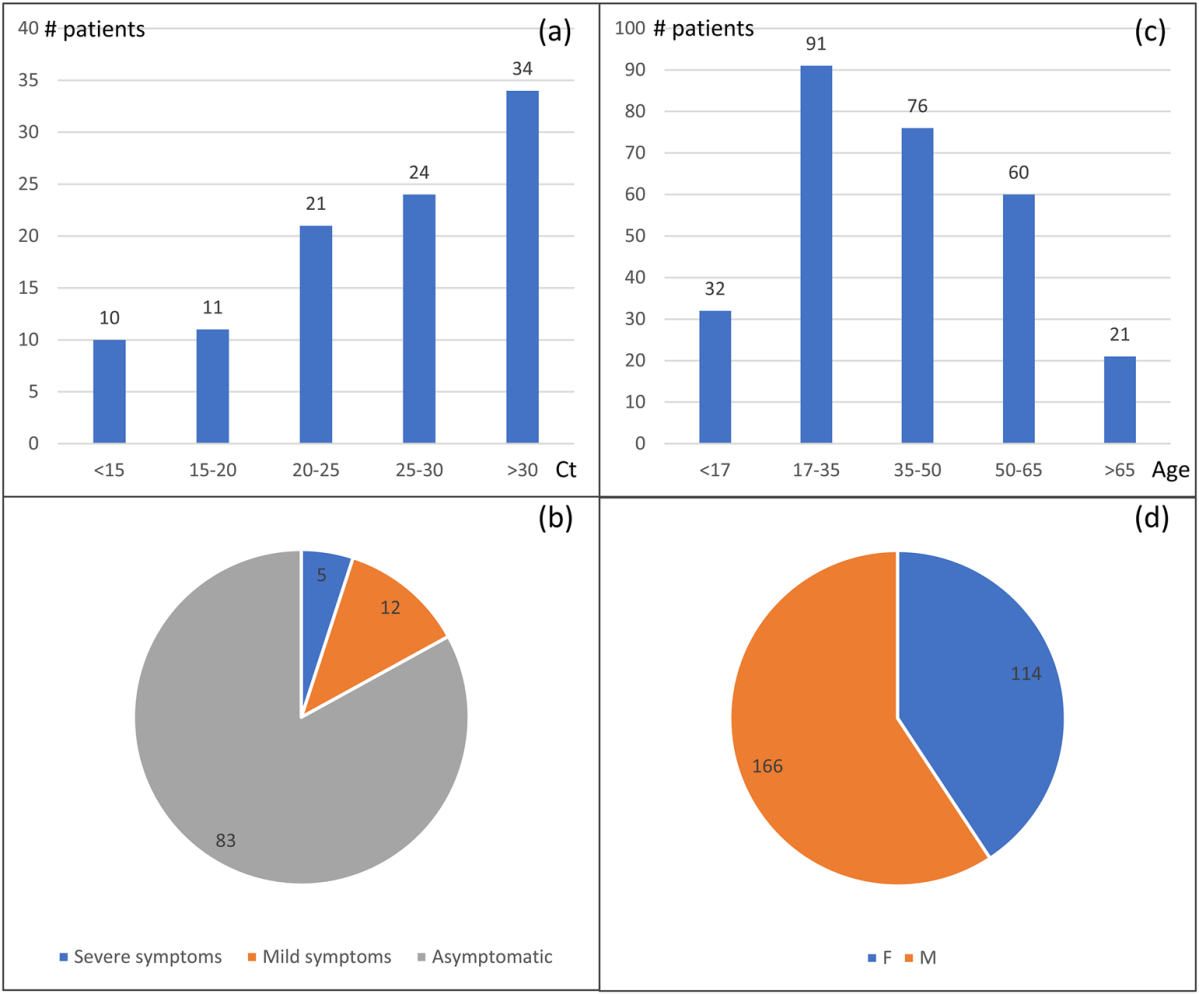
Clinical data of Covid-19 tested patients. (**a**) Ct values distribution based on the number of samples (**b**) Distribution of symptomatic and asymptomatic positive patients, (**c**) Distribution of patients by age. (**d**) Distribution of patients by sex.

In this study, 280 RNA extracts from nasopharyngeal samples collected from 280 Moroccan patients are used to train, test, and validate our classification models (Fig. 2). These samples were collected from 100 SARS-CoV-2 PCR positive patients and 180 SARS-CoV-2 PCR negative patients with ages ranging from 11 to 67 years old. Cycle threshold (Ct) values of positive samples ranged from 11.7 to 34 with an average of 25.6 and a median of 26.1. 17 Covid-19 patients were experiencing symptoms while the remainder of the tested cohort was asymptomatic.

Nasopharyngeal samples specimens were collected using swabs. They were immediately inserted into sterile tubes containing 1–3 mL of viral transport media. We used extraction kits based on the magnetic beads method, followed by washing steps then elution. 100 μL of viral transport media were added to the preloaded kit, while the remaining purification process was fully automated by the extractor in Viral Mode. The samples output is of 50 µL. These protocols were based on the manufacturer’s recommendation. The real-time PCR assay was then performed using the Takyon Real-Time One-Step RT-PCR Master Mix and Eurogenetic kit for covid-19 E-gene (see Table 1, Supplementary Information). The RNA quality of the samples was determined by the optical method of absorbance in the ultraviolet. For nucleic acids, the three main wave numbers of interest are 260 nm, 280 nm and 230 nm. The ratio of the absorbance at 260 versus 280 nm (A260/280) is generally used to assess the purity of nucleic acids as well as the DNA *vs* RNA ratio. Absorbance at 280 nm is indicative of proteins content in the sample. Measurements at 230 nm were used to determine the amounts of other contaminants that may be present in the samples, such as guanidine thiocyanate and guanidine hydrochloride, common in nucleic acid purification kits (see Supplementary Information).

**Table 1:** Comparison of predicting performance on the testing set using Logistic Regression.

Spectral region (cm^−1^)	Spectra signal	Accuracy (%)	Sensitivity (%)	Specificity (%)	AUC (× 100)	Accuracy (%)	Sensitivity (%)	Specificity (%)	AUC (× 100)
		Out-of-sample				In-sample			
600 to 4500	Raw	95.7	94.1	96.7	98.4	100	100	100	100
	Baseline Corrected	96.8	94.1	98.3	99.8	100	100	100	100
	1st Der	96.8	97	98.3	99.9	100	100	100	100
	2nd Der	97.8	97	98.3	99.1	100	100	100	100
900 to 1800	Raw	83.1	70.5	86.5	89	92.1	82.3	100	94.3
	Baseline Corrected	84.2	73.5	86.5	90.5	94.7	88.2	100	95.5
	1st Der	93.6	94.1	92.4	97.7	100	100	100	100
	2nd Der	94.7	94.1	95.4	97.2	100	100	100	100

### FTIR and machine learning

**Figure 3:**
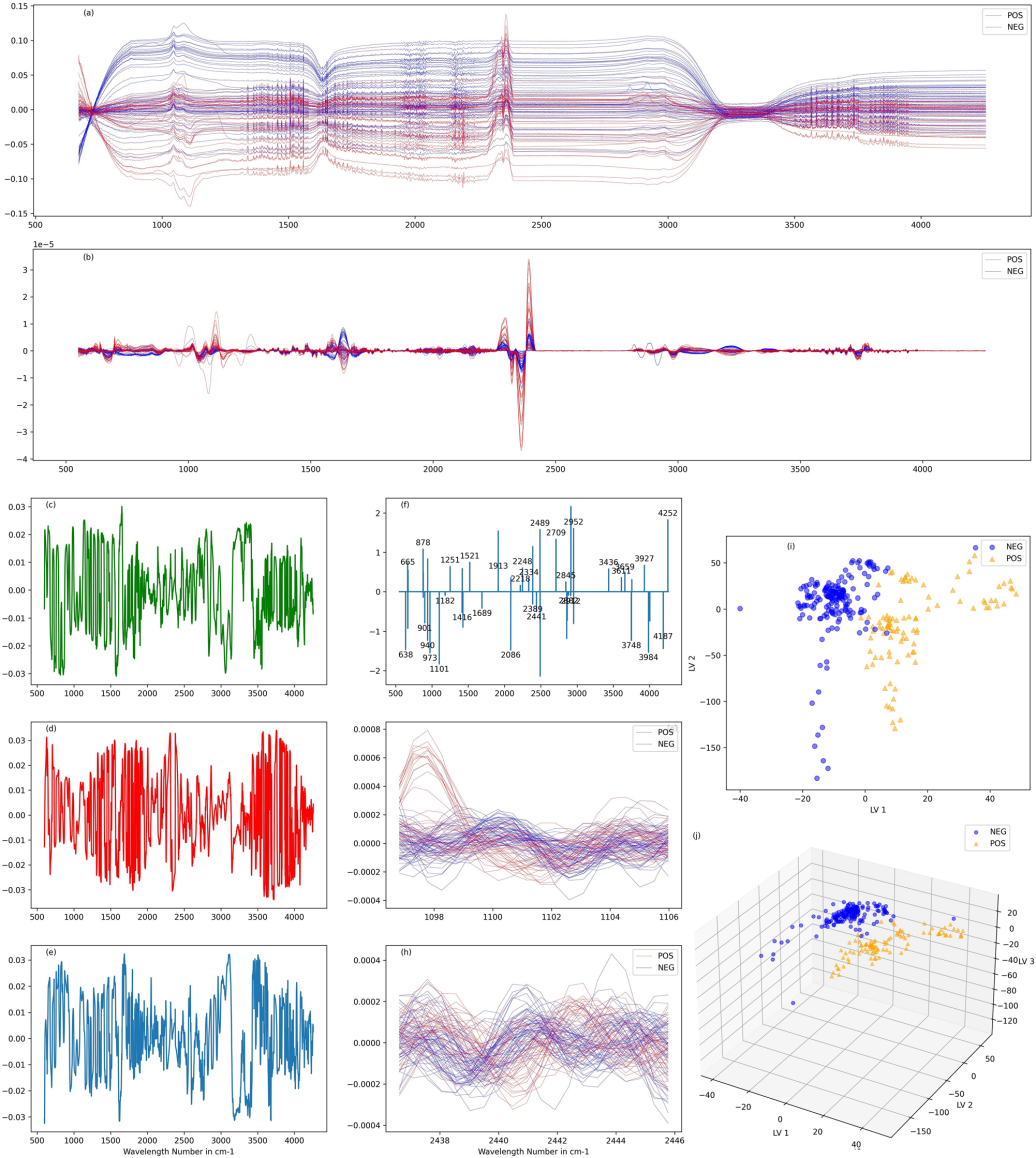
Detection of SARS-CoV-2 with multivariable analysis. (**a**) Raw Spectra (**b**) Sample of 2nd derivative of Savitzky–Golay smoothened spectra of positive and negative samples. (**c**, **d**, **e**) First three latent variables of PLS-DA. (**f**) Coefficients of variables selected by sparse classification algorithm of second derivative of raw spectra (**g**, **h**) Zooms on regions indicated by sparse classification. (**i**)Projection of the 280 spectra used according to the first two latent variables obtained. (**j**) Projection of the 280 spectra used according to the first three latent variables obtained.

Primary analysis of the vibrational chemical bonds, shape and band intensity on the raw recorded spectra (Fig. 3a) did not give any useful information on the Covid-19 status of the patients, and no details can be extracted even after in-depth investigation of baseline corrected spectra. We then apply machine learning algorithms to the spectra and compare their out-of-sample performance on the testing set (Fig. 3).

We apply discrete second derivative of the raw spectra acquired (Fig. 3b). We center and normalize the data of the transformed spectra. We finally use machine learning algorithms to build classification models. We also evaluate other methods such as first derivative and found that best results are achieved using first and second derivative (see Supplementary Information). We report a sparse solution for sparse logistic regression in Fig. 3f, that stress out relevant wavenumbers in our classification method.

Given the measurement’s sampling interval in this region (~0.48 cm^−1^), a total of 8287 features (variables) were included for the 280 samples. Due to the unbalanced ratio between the number of samples (280) and the number of variables (~ 8200), we use dimension reduction techniques, namely principal component analysis (PCA), partial least square (PLS) and Sparse Classification^36^ and then use logistic regression for PCA and PLS and support vector machine (SVM) and kernel SVM for classification.

To improve interpretability, we also use the sparse classification approach. Classic methods such as PCA and PLS build principal components and latent variables using all available variables, which hinders their interpretability (Fig. 3c,d,e). Here we also build classification models using Mixed Integer Optimization based classification algorithms. These methods build a classification model using a relatively low number of variables, much lower than the number of the features of the spectra, to improve interpretability (Fig. 3f). Given a data matrix X (representing spectra in our case), a response vector Y (here positive or negative outcome), a loss function ℒ and a regularization function π, sparse classification algorithms build classification models by solving the following type of problem:

$$\begin{aligned} & {\text{Min}}_{\upbeta } {\mathscr{L}}({\text{Y}},{\text{X}},{\upbeta }) + \upgamma \uppi (\upbeta ) \\ & {\text{s.t}}.\quad \|\upbeta \|_{0} \le {\text{ k}} \\ \end{aligned}$$

where γ is a non-negative parameter, k a positive integer and ||.||_0_ is the L_0_ norm indicating the number of non-zero variables in β. In this work we use a sigmoid function as a loss function, which is identical to logistic regression, and Tikhonov regularization. Regularization provides robustness to the model built *vs* noise. Tikhonov regularization is particularly efficient in addressing normal noise and has proven to be particularly efficient in spectroscopy applications^37^.

Dimension reduction methods project the data into a lower dimension space that is believed to allow a strong predictive power. Thus, they bolster the performance of the downstream binary classification methods. We use of cross-validation to tune the hyperparameters of these methods. For the performance evaluation, we use, as a standard practice, 67% of the samples for training and the remaining for testing. We randomly choose 185 samples for training and 95 for testing. In Supplementary Information, we also assess statistical significance by randomly shuffling 25 times the training/testing samples and report tests results in Table 1. All binary classification models are evaluated based on their ability to discriminate the outcome of interest. We present standard deviation for all reported metrics in Supplementary Information. We report AUC and its standard deviation for all methods in Supplementary Information. This metric captures the information about the model performance independently of the detection threshold selected. Moreover, we report the accuracy, sensitivity and specificity (see Supplementary Information for a full comparison of algorithms’ respective performance).

We observed that the choice of spectral region and data processing technique play a key role in the performance of the models. Indeed, expanding the spectral region from the classic bio fingerprint region of 1800–900 cm^−1^ to 600-4500 cm^−1^ boosts the accuracy by up to 12.6% (in the case of raw spectra). Although less important, there are also benefits using signal processing transformations. When 600–4500 cm^−1^ region is chosen, the improvement reported is 1.9%. The second derivative seems to offer the highest performance and improves the stability of the algorithms (see Supplementary Information). The choice of classification algorithm is less important (we provide a full discussion in Supplementary Information). The highest results were achieved using second derivative of captured spectra and the PLS-based methods and Logistic regression achieve an accuracy of 97.8%, a sensitivity of 97%, and specificity of 98.3%.

The biomarkers depicted by the sparse model (which results from an unsupervised method) are largely discussed in the FTIR spectroscopy literature, the most representative are reported in Table 2.

**Table 2:** Tentative assignment of wavenumber markers used by sparse classification.

Wave number (cm^−1^)	Tentative Assignments^38–42^
638, 665	Guanine breathing mode
878	Out-of-plane vibrations of nucleobases
901, 940	Ribose phosphate backbone
973	C–O and C–C ribose
1101	Symmetric stretching P–O–C, RNA ν(C–O) ribose
1182	C–O and phosphate vibrations
1251	P = O, PO_2_^−^asym
1416	Stretching C–N, N–H and C–H deformation
1521	C nucleobase both in RNA ss or ds, amide II
1689	C_6_=O_6_ of G in ds, Amid I, C=O Guanine and N–H deformation, ν_asym_(C_2_=O) vibration in RNA
1913, 2086	bands of second order
2218, 2248, 2334, 2389, 2441, 2489, 2709, 2845, 2882, 2952	Stretch C–H, N–H
3611, 3436, 3659, 3748, 3927	Stretch N–H asym and O–H asym

Careful analysis of the maximum footprint spectral region of the RNAs spectra for the positive and negative samples of SARS-CoV-2 RNAs (Fig. 3f), indicates the presence of three main visible domains: one located at 600–1350 cm^−1^, the other at 1500–1700 cm^−1^ and at 2300–3900 cm^−1^. The first domain is attributed to the phosphate backbone vibrations (νP-O) with the 1000–1182 cm^−1^ region arising from symmetric stretching vibrations of PO_2_^−^ and assigned to the νC-O stretching vibration of the phosphodiester and the ribose. In addition, the spectral region 1200–1300 cm^−1^ could be attributed to PO_2−_ asymmetric stretching vibration of the RNA usually centered at the 1251 cm^−1^. The third 1500–1700 cm^−1^ region could be assigned to RNA nucleobases. Furthermore, this region overlaps with a series of biomarker bands usually ascribed to Amid I and II vibrations. The region 2400–3900 cm^−1^ is in line with the stretching vibrations of OH, NH, and CH groups. Taken together, these data are clearly in accordance with the RNA signature confirming the robustness of the FT-IR/machine learning dual coupling in virus detection and patient classification.

In order to assess the specificity of the technique towards other viruses and its limit of detection (LoD) we investigate a set of samples and analyze them using FTIR spectroscopy and machine learning.

### Specificity towards other viruses

We use a total of 123 samples including 31 samples featuring the entire genome of SARS-CoV-2 with various concentrations as well as samples including the entire genome of each of human bocavirus 1, human coronavirus 229E, human coronavirus NL63, human coronavirus OC43, human enterovirus 68, human parainfluenza virus 1, human parainfluenza virus 4, rhinovirus 89, influenza A, influenza B, influenza H3N2measles, MERS coronavirus mumps and SARS-CoV-1 (2 samples each with 500 copies/μl concentration). We also performed experiments on42 PCR positive controls containing four individual non-infectious DNA plasmids coding for the RdRp gene, the E gene, the N gene, the RNAse P gene and 20 DEPC-treated water samples. The concentration of SARS-CoV-2 samples vary between 10,000 copies/μl and 0.5 copies/μl. A full description is provided in Table 3.

**Table 3:** Synthetic RNA viruses used to assess the specificity. (**a**) Anti-sense strand, (**b**) sense strand.

Name	Accession	Virus Type	Length
Twist synthetic Influenza H1N1 (2009)	NC_026431, NC_026431, NC_026431, NC_026431, NC_026431, NC_026431, NC_026431	ssRNA (−)^a^	13,158
Twist synthetic Influenza H3N2	NC_007366, NC_007367, NC_007368, NC_007369, NC_007370, NC_007371, NC_007372, NC_007373,	ssRNA (−)	13,627
Twist synthetic Influenza B	NC_002204, NC_002205, NC_002206, NC_002207, NC_002208, NC_002209, NC_002211	ssRNA (−)	14,452
Twist synthetic human Bocavirus	MG953830.1	ssDNA	5164
Twist synthetic human Enterovirus 68	NC_038308.1	ssRNA (+)b	7367
Twist synthetic human Rhinovirus 89	NC_001617.1	ssRNA (+)	7152
Twist synthetic Mumps virus	NC_002200.1	ssRNA (−)	15,384
Twist synthetic human Parainfluenza virus 1	NC_003461.1	ssRNA (−)	15,600
Twist synthetic Measles virus	NC_001498.1	ssRNA (−)	15,894
Twist synthetic human Parainfluenza virus 4	NC_21928.1	ssRNA (−)	17,052
Twist synthetic human Coronavirus 4	NC_0022645.1	ssRNA (+)	27,317
Twist synthetic human Coronavirus NL63	NC_005831.2	ssRNA (+)	27,553
Twist synthetic human Coronavirus OC43	NC_006213.1	ssRNA (+)	30,741
Twist synthetic human Coronavirus Tor 2	NC_004718.3	ssRNA (+)	29,751
Twist synthetic human Coronavirus 2c EMC/2012	JX869059.2	ssRNA (+)	30,119

We recorded 64 spectra for each sample using the same parameters and equipment as those used for the clinical samples (Table S2) and then averaged the 64 spectra for each sample. We then apply the second derivative of the average spectra in the 1800–900 cm^−1^ region which resulted in 1867 variables. We first disregard the SARS-CoV-2 samples of less than 25 copies/μl to assess the specificity. 100% accuracy is achieved on the resulting set using sparse classification algorithm in-sample. The separation is achieved using only 13 variables out of the 1867 variables constituting the spectra in the 1800-900 cm^−1^ region. 2 pairs of these variables are adjacent and overlap in the spectra, which indicate that only 11 frequencies could be used to separate the set. The number of data points (109 spectra) is almost one order of magnitude lower than the number of dimensions used to classify. We enumerate the frequencies and provide a tentative assignment for the attribution of the specific wavenumbers (Table 4). Remarkably, we found that the set of variables used in this model and the set of variables used for clinical samples are disjoint, which indicates that the viral signature could be found in various variables settings. The variables used by the sparse algorithm for clinical samples can also be used to separate the positive synthetic samples from the synthetic negative ones.

**Table 4:** Variables used to separate positives and negative samples with 100% accuracy.

Wave number (cm^−1^)	Tentative assignments^38–42^
1038	ν(C–O) ribose. symmetric stretching P–O–C
1074	ν_asym_(PO_2_^−^) symmetric and ν (C-O) ribose
1131 and 1172	RNA ν (C=O). ribose
1172 and 1174	ν (C-O) and phosphate vibrations (non-H-bonded mode of C–OH) ^x^
1210	ν_asym_ (PO_2_^−^)
1640. 1670. 1673	Amid I.ν_a_ (C_2_=O).ν_asym_(C_5_=O).ν_asym_ (C_6_=O). can overlap with water (ν_2_)
1760	ν carbonyl stretching

It is worth noting that the 11 frequencies used for the separation are in line with the RNA signature (Table 4). These frequencies can be classified into two spectral regions. The first one, 1038–1220 cm^−1^, is typically assigned to the fingerprint of the sugar and the phosphate backbone of the RNA. Indeed, 1038 and 1074 cm^−1^ bands could be assigned to the (C–O) ribose and P–O–C symmetric stretching at position 5 of the ribose. 1172 and 1174 cm^−1^ are ascribed to the single C–O bond vibrations of the ribose and phosphate as well as to the free hydroxy groups at 2’ position. 1210 cm^−1^ frequency is mainly ascribed to ν (PO_2_^−^) asymmetric vibration. The second region, 1640–1760 cm^−1^, can be attributed to in-plane vibrations of nucleobases related to double bonds in the 5 and 6 member rings heterocycles: C=C, C=N and C=O of purine (A. G) and pyrimidines (C. U). 1640–1673 frequencies are in line with vibrations of amid I, ν_a_ (C_2_=O), ν_a_ (C_5_=O) and ν_a_ (C_6_=O). Strong band at 1760 cm^−1^ is a typical marker of carbonyl groups.

To assess out-of-sample performance, we use Leave-one-out technique. We iteratively remove the spectrum for each sample of human bocavirus 1, human coronavirus 229E, human coronavirus NL63, human coronavirus OC43, human enterovirus 68, human parainfluenza virus 1, human parainfluenza virus 4, rhinovirus 89, influenza A, B and H3N2 measles, MERS coronavirus, mumps and SARS-COV-1 and train with remaining 108 spectra. The algorithm predicts correctly 25 out of the 30 samples presented (2 of each virus). The five samples that are predicted as false positives are one sample of each of influenza A and B, MERS, coronavirus OC43, and enterovirus 68. We also note that the training set is relatively limited regarding the variety of viruses. Undoubtedly, the out of sample specificity might be improved with a richer training set, i.e., by increasing the number of samples in the training set.

### Limit of detection

The limit of detection (LoD) is a measure of the analytic sensitivity of the method, regarding the lowest copy number detected 95–100% of the time. Its quantification is highly dependent on the technique used, its accuracy, the kits’ specification and the component to be analyzed^43^. Regarding the quantification of LoD for SARS-CoV-2 detection, several units were reported such as copies/ml, copies/μl, copies/reaction volume, mol/l making the comparisons between the techniques difficult^44,45^. In our case we used a copies/μl as the correlation between Ct and the concentration (copy/μl) is easier to handle^46^.

For this study, we train sparse classification algorithm using the samples with concentration higher than 25 copies/μl and test on the remaining samples (Table 5). We achieve100% accuracy of all samples with concentrations as low as 10 copies/μl. In addition, we observe a weak detection with concentrations below 10 copies/μl on synthetic samples, which is consistent with the results obtained on clinical samples. Indeed, clinical samples with Ct of 31 and higher are correctly diagnosed. This level of Ct is equivalent to a number of copies close to 10 copies/μl^44^. It is worth noting that all clinical samples with a Ct higher or equal to 32, which corresponds to the limit of our RT-PCR set-up, are correctly diagnosed by our technique.

**Table 5:** Detection limit: distribution of predictions on positive samples after training depending on samples’ concentration.

Concentration (copies/ul)	Number of samples	Number of correct predictions
25	3	3
10	3	3
5	3	1
3	3	0
0.5	5	0

## Discussion

During the last centuries, the most severe and devastating pandemics, with high mortality rate, have been mainly ascribed to RNA viruses. Presently, the world is experiencing an unprecedented health crisis due to a new RNA virus, SARS-CoV-2 (Covid-19). This pandemic still has dramatic effects with devastating economic, social and health consequences. The diagnosis of SARS-CoV-2 and the control of its dissemination raised serious questions in developing countries. While no efficient treatment is now available, the mass diagnosis of populations during the next months is crucial to limit the spread of Covid-19. Many countries are still trying to scale up diagnostic screening tests to meet the high demand. Undoubtedly, there is an urgent need for an effective and appropriate diagnostic test for all populations to control the spread and minimize its devastating consequences.

To address these issues, we demonstrate in this work that the use of FT-IR spectroscopy on RNA extracts and machine learning is highly convenient and appropriate for the diagnosis of Covid-19 disease. Indeed, 280 symptomatic and asymptomatic-suspected Moroccan patients with specific clinical indications were tested for their Covid-19 status using swab samples. Among the samples of the 280 patients, 100 were Covid-19 positives and 180 negatives by quantitative RT–PCR assay used as a control. The extracted RNAs were then analyzed by ATR infrared spectroscopy and the obtained spectra were used to train and test a machine learning classification method. The machine learning was then applied and showed very promising results with 97.8% accuracy, 97% sensitivity and 98.3% specificity. The FTIR spectra indicates the presence of three main visible domains located at 600–1350 cm^1^, at 1500–1700 cm^−1^ and at 2300–3900 cm^−1^ clearly ascribed to RNA fingerprint, i.e., the phosphate backbone vibrations (νP-O), the νC-O stretching vibrations of the ribose sugar and the specific RNA nucleobases. The region 2400–3900 cm^−1^ is attributed to the stretching vibrations of OH, NH, and CH group.

To get further insight into the selectivity of this technique against other viruses, we used 123 samples including 31 samples featuring the entire genome of SARS-COV-2 as well as samples including the entire genome of human bocavirus, human coronaviruses (229E, NL63,OC43, MERS, SARS-CoV), enterovirus 68, human influenza viruses (A, B, H3N2), human parainfluenza viruses(1 and 4) and rhinovirus 89. We found that this technique is 100% in agreement with RT-PCR used a standard control, reaching high viral selectivity even with the high structural similarities of the RNA viruses. Furthermore, the limit of detection was assessed using both synthetic and clinical samples with different concentrations, from 0.5 to 25 copies/μL. The LoD was determined to be 10 copies/μL for both samples.

A remarkable work was just published by Martin and Coll^47^. This work uses ATR-FTIR on a saliva matrix instead of RNA extract. This report is highly interesting and complementary to the present work for comparison studies, i.e., saliva matrix versus RNA extract. Indeed, the performances observed from saliva are less than those observed with the RNA extracts in terms of sensitivity (97 vs 95%) and specificity (98.3% vs 89%), the accuracy for RNA extract is high 97.8% while it is not reported for the saliva. In other words, saliva swab is a complex medium containing complex cellular materials including phospholipids, viral and non-viral nucleic acids and proteins, etc. making the repeatability inter-patients difficult and the interpretability of spectra. The use of extracted viral RNA has several advantages in terms of purity (one major component) allowing clear signature, high correlation and interpretability of the FT-IR spectra.

Furthermore, the covid-19 diagnosis community is working on developing new techniques to increase testing capacity, overcome limitations (such as the use of reagents) and shorten the testing time. Each technique requires resources (equipment, reagents, etc.) that may not overlap creating thus the possibility to expand the testing capabilities. The multiplicity of testing techniques can be an answer to the testing challenge that World population is facing. The performances of FTIR-based technique are comparable to other techniques developed by the scientific community (Table 6).

**Table 6:** Characteristics of different Sars-CoV-2 detection techniques. (a) Authors' estimates. (b) The matrix solution was prepared with α-cyano-hydroxy-cinnamic acid (CHCA) at 1% in acetonitrile/0.1% trifluoroacetic acid (1:1). c) RT-PCR Limit of Detection in copies/mL^44^.

	ATR-FTIR	CRISPR–Cas12-based^22^	MALDI-MS^48^	RT-PCR
Test Ct range	11 to 39	20 to 37	16 to 37	–
Accuracy	98%	98%	94%	–
Sensitivity	97%	95%	95%	–
Specificity	98%	100%	93%	–
LoD (in copies/μl)	10	10	NA	10 to 511^c^
Sample-to-result time (min)	21	30–40	25-30^a^	120
Consumables	RNA extraction kits	RNA extraction & Crisper kits	CHCA^b^	RNA extraction & PCR kits
Equipment	FTIR Spectrometer, RNA extractor	RT-Lamp, Lateral flow Strip, RNA extractor	Mass Spectrometer	PCR machine, RNA extractor

The FTIR spectroscopy shows a promising potential for mass testing since it is reagent free, fast and provides strong predicting performances. The models developed in this study provide 97% sensitivity and could enable the use of FTIR as a tool that could filter samples going through PCR, which could expand the testing capabilities, especially in developing countries where the testing capacity is saturated. All laboratories performing RNA extraction (virtually all PCR testing centers) could benefit from this technique, which would cut post-RNA testing time from hours to minutes and could boost testing capacity whenever RNA extraction capacity is under-utilized relatively to PCR. This technology could also enable epidemiologic surveillance. It can be adapted not only in clinical laboratories and hospitals but also in several locations as well (airports, schools and universities, etc.) using a simple portable monitoring FT-IR device.

In summary, the combination of FT-IR and machine learning allows quantitative predictive agreement and classification of both positive and negative samples of SARS-CoV-2 in a faster manner compared to RT-PCR (1 to 1.5 min vs. 2–5 h) with 97.8% accuracy for the detection of this coronavirus in 280 patient samples. Compared to the RT-PCR requiring the enzymes and amplification kits, the FTIR spectroscopic method presented here is reagent-free and can be used in point of care with limited facilities. Finally, the use of RNA extracts and FTIR spectroscopy could also be considered in the diagnosis of other viruses.

## Materials and methods

### Samples collection and RNA extraction

Nasopharyngeal swab specimens were collected using only swabs with a synthetic tip. Swabs were immediately inserted into sterile tubes containing 1–3 ml of viral transport media. In this study, total RNA was extracted using four different nucleic acid extractors from different vendors Amplix (ZP01001), Molarray (MA-32T), Bioer (NPA-32P) and Genrui (v3-NE48/96). For all four methods, viral transport media was added to the preloaded Kit (Bioer Bsc86 Magabio Plus Virus DNA/RNA Purification Kit III, Genrui nucleic acid extraction kit NE-01A and 3150001 Amplix viral Nucleic acid extraction kit), while the remaining purification process was fully automated by corresponding extractor in Viral Mode. These protocols are all dedicated to viral RNA extraction and based on a magnetic beads method which ensure a good quality of total RNA after quantification. Extractions were performed according to the manufacturer's guidelines.

### RT real time PCR

Real-time RT-PCR assay was performed using the Takyon Real-Time One-Step RT-PCR Master Mix and Eurogenetic kit for both E and RdRp SARS-CoV-2 genes as per the protocol below:

Each 25 μL reaction mixture contained 12.5 μL of 2X reaction buffer, 1 μL of forward and reverse primers at 10 mM, 0.5 μL of probe at 10 mM, 0.25 μL of RTenzyme, 0.5 μL RNase inhibitor and 5 μL of total extracted RNA with a concentration of 2 ng/ul or higher. Amplification was carried out in 96-well plates on QuantStudio1 machine. Thermocycling conditions consisted of 55 °C for 10 min for reverse transcription, followed by 95 °C for 3 min and then 45 cycles of 95 °C for 15 s and 58 °C for 30 s. Each run included one SARS-CoV-2 positive control and one template control. For a routine workflow, the E gene assay was carried out as the first-line screening indicator followed by confirmatory testing with the RdRp gene assay. This assay is setup with the same conditions as the E gene. Both E and RdRp screening assays were performed according to the manufacturer’s guidelines.

### ATR-FTIR spectroscopy

We use ATR-FTIR spectroscopy (Attenuated Total Reflection Fourier Transform Infrared) to collect spectra. ATR-FTIR spectra were acquired using Jasco 4600 ATR-FTIR spectrometer with a deuterated lanthanum α-alanine doped triglycine sulphate (DLaTGS) pyroelectric detector. The detector is operated with temperature stabilization using electrical peltier temperature control. The spectrometer is paired with a high-intensity ceramic light source. We perform single reflection ATR using high-throughput monolithic diamond crystal and 64 spectra are averaged. We apply the torque-limiter pressure applicator for reproducible sample pressure contact for sample measurements. We use distilled water as solvent background. We spread 3 μL of each sample on the ATR crystal, ensuring that no air bubbles were trapped. We do not dry samples on the spectra to simplify sample manipulation and decrease the testing time, at the expense of having to deal with the absorption from water. After the acquisitions, we clean the crystal with ethanol (70% v/v) and dry it using paper towel (see Supplementary Information). We collect the 600–8000 cm^−1^ region with a spectral resolution of 2 cm^−1^. We use 600 cm^−1^ to 4500 cm^−1^ (Table 1) as a representative region in our study, with a special focus on the traditional 900 to 1800 region (Fig. 3), known as the RNA bio fingerprint region^28^.

### Machine learning

We used mainly publicly available libraries on a commercial personal computer. PCA, PLS, Logistic regression, SVM, Kernel SVM and Discriminant analysis were all solved using “sklearn” v0.23 running on Python 3.7.3. We use “SubsetSelection” package on Julia language to perform sparse classification; warm-starting the algorithm with a good solution is key to reduce the computational time. We use the “signal” library for signal processing on Python 3.7.3. A quarter of the training data was used for cross-validation to tune the hyperparameters of the algorithms used. The computer used is has an Intel CORE i7-8750H CPU at 2.2 GHz with 16 GB RAM running on Windows 10.

### Ethical and biosafety statement

In this work all methods were carried out in accordance with relevant guidelines and in line with the Moroccan’ regulations. All experimental protocols were approved by Institutional Ethic Committee UM6P-Anoual. Informed consent was obtained from all subjects and also, for subjects under 18, informed consent from a parent and/or legal guardian. No identity of any sample was related to the name of the patient or other information that could lead to personal identification. Specific patient information was limited to the present work (sex, age and symptoms). All samples used for RT-PCR were split for FT-IR analyses and used according to Laboratory protocols. All samples were deactivated before RNA extraction in a biosecurity Lab. All materials were cleaned before and after each experiment to prevent any source of contamination. All consumables and Covid waste are managed by a private company and incinerated according to the authorities’ guidelines.

## Supplementary Information

Supplementary Information.

## Data Availability

All data needed to evaluate the conclusions in the paper are present in the paper. Additional data related to this paper may be requested from the authors.

## References

[1] Nickol ME, Kindrachuk J (2019). A year of terror and a century of reflection: Perspectives on the great influenza pandemic of 1918–1919. BMC Infect. Dis..

[2] Taubenberger JK, Morens, (2006). 1D. M. 918 Influenza: The Mother of All Pandemics. Emerg. Infect. Dis..

[3] Hemelaar J (2013). Implications of HIV diversity for the HIV-1 pandemic. J. Infect..

[4] Manns MP, Buti M, Gane E, Pawlotsky JM, Razavi H, Terrault N, Younossi Z (2017). Hepatitis C virus infection. Nat. Rev. Dis. Prim..

[5] Fedson DS (2018). What treating Ebola means for pandemic influenza. J. Public Health Policy..

[6] Fineberg HV (2014). Global health: Pandemic preparedness and response - Lessons from the H1N1 influenza of 2009. N. Engl. J. Med..

[7] Reperant LA, Osterhaus ADME (2017). AIDS, Avian flu, SARS, MERS, Ebola, Zika… what next?. Vaccine.

[8] Zhou F (2020). Clinical course and risk factors for mortality of adult inpatients with COVID-19 in Wuhan, China: A retrospective cohort study. Lancet.

[9] Pascarella, G., Strumia, A., Piliego, C., Bruno, F., Del Buono, R., Costa, F., Scarlata & Agrò, S. F. E. COVID-19 diagnosis and management: a comprehensive review. *J. Intern. Med. 288*, 192–206 (2020). 10.1111/joim.13091 PMC7267177 32348588

[10] Yuki K, Fujiogi M, Koutsogiannaki S (2020). COVID-19 pathophysiology: A review. Clin. Immunol..

[11] WHO, No Title, p. WHO Coronavirus Disease (COVID-19) Dashboard, released Friday February 5th 2021.

[12] Lone SA, Ahmad A (2020). COVID-19 pandemic—An African perspective. Emerg. Microbes Infect..

[13] Gilbert G, Pullano G, Pinotti F, Valdano E, Poletto C, Boëlle PY, D’Ortenzio E, Yazdanpanah Y, Eholie SP, Altmann M, Gutierrez B, Kraemer MUG, Colizza V (2020). Preparedness and vulnerability of African countries against importations of COVID-19: A modelling study. Lancet.

[14] Chakrabortya S, Mallajosyulab V, Tato CM, Tan GS, Wang TT (2021). SARS-CoV-2 vaccines in advanced clinical trials: Where do we stand?. Adv. Drug Deliv. Rev..

[15] Graham BS (2020). Rapid COVID-19 vaccine development. Science.

[16] Sharma O, Sultan AA, Ding H, Triggle CR (2020). A review of the progress and challenges of developing a vaccine for COVID-19. Front. Immunol..

[17] Le Thanh T, Andreadakis Z, Kumar A (2020). The COVID-19 vaccine development landscape. Nat. Rev. Drug. Discov..

[18] Starr TN, Greaney AJ, Addetia A, Hannon WW, Choudhary MC, Dingens AS, Li JZ, Bloom JD (2021). Prospective mapping of viral mutations that escape antibodies used to treat COVID-19. Science.

[19] Anderson EJ, Rouphael NG, Widge AT (2020). Safety and immunogenicity of SARS-CoV-2 mRNA- 1273 vaccine in older adults. N. Engl. J. Med..

[20] Voysey, M., Clemens, S. A. C, Madhi, S. A. et al. Safety and efficacy of the ChAdOx1 nCoV-19 vaccine (AZD1222) against SARS-CoV-2: an interim analysis of four randomised controlled trials in Brazil, South Africa, and the UK. *The Lancet* (2020) https://www.thelancet.com/journals/lancet/article/PIIS0140-6736(20)32661-1/abstract. 10.1016/S0140-6736(20)32661-1 PMC7723445 33306989

[21] Udugama B, Kadhiresan P, Kozlowski HN, Malekjahani A, Osborne M, Li VYC, Chen H, Mubareka S, Gubbay JB, Chan WCW (2020). Diagnosing COVID-19: The disease and tools for detection. ACS Nano.

[22] Wölfel R, Corman VM, Guggemos W, Seilmaier M, Zange S, Müller MA, Niemeyer D, Jones TC, Vollmar P, Rothe C, Hoelscher M, Bleicker T, Brünink S, Schneider J, Ehmann R, Zwirglmaier K, Drosten C, Wendtner C (2020). Virological assessment of hospitalized patients with COVID-2019. Nature.

[23] Bordi L, Piralla A, Lalle E, Giardina F, Colavita F, Tallarita M, Sberna G, Novazzi F, Meschi S, Castilletti C, Brisci A, Minnucci G, Vettamanzi V, Baldanti F, Capobianchi MR (2020). Rapid and sensitive detection of SARS-CoV-2 RNA using the Simplexa^TM^ COVID-19 direct assay. J. Clin. Virol..

[24] Sun B, Feng Y, Mo X, Zheng P, Wang Q, Li P, Liu X, Chen Z, Huang H, Zhang F, Luo W, Niu X, Hu P, Wang L, Peng H, Huang Z, Feng L, Li F, Zhang F, Li F, Zhong N, Chen L (2020). Kinetics of SARS-CoV-2 specific IgM and IgG responses in COVID-19 patients. Emerg. Microbes Infect..

[25] Cheong J, Yu H, Lee CY (2020). Fast detection of SARS-CoV-2 RNA via the integration of plasmonic thermocycling and fluorescence detection in a portable device. Nat. Biomed. Eng..

[26] Reijns MAM, Thompson L, Acosta JC, Black HA, Sanchez-Luque FJ, Diamond A (2020). A sensitive and affordable multiplex RT- qPCR assay for SARS-CoV-2 detection. PLoS Biol.

[27] Ganguli A, Mostafa A, Berger J, Aydin MY, Sun F, Stewart de Ramirez SA, Valera E, Cunningham BT, King WP, Bashir R (2020). Rapid isothermal amplification and portable detection system for SARS-CoV-2. PNAS.

[28] Nouri R, Tang Z, Dong M, Liu T, Kshirsagar A, Guan W (2021). CRISPR-based detection of SARS-CoV-2: A review from sample to result. Biosens. Bioelectron..

[29] Guo L, Sun X, Wang X, Liang C, Jiang H, Gao Q, Qu B, Fang S, Mao Y, Chen Y, Feng G, Gu Q, Wang RR, Zhou Q, Li W (2020). SARS-CoV-2 detection with CRISPR diagnostics. Cell Discov..

[30] Broughton, J. P., Deng, X., Yu, G., Fasching, C. L., Servellita, V., Singh, J., Miao, X., treithorst, J. A., Granados, A., Sotomayor-Gonzalez, A., Zorn, K., Gopez, A., Hsu, E., Gu, W., Miller, S., Pan, C. Y., Guevara, H., Wadford, D. A., Chen, J. S. & Chiu, C. Y. CRISPR–Cas12-based detection of SARS-CoV-2. *Nat. Biotechnol. 38*, 870–874 (2020). 10.1038/s41587-020-0513-4 PMC9107629 32300245

[31] Liu R, Han H, Liu F, Lv Z, Wu K, Liu Y, Feng Y, Zhu C (2020). Positive rate of RT-PCR detection of SARS-CoV-2 infection in 4880 cases from one hospital in Wuhan, China, from Jan to Feb 2020. Clin. Chim. Acta..

[32] Yan Y, Chang L, Wang L (2020). Laboratory testing of SARS-CoV, MERS-CoV, and SARS-CoV-2 (2019-nCoV): Current status, challenges, and countermeasures. Rev. Med. Virol..

[33] Santos MCD, Morais CLM, Nascimento YM, Araujo JMG, Lima KMG (2017). Spectroscopy with computational analysis in virological studies: A decade (2006–2016). TrAC - Trends Anal. Chem..

[34] Fernandes, J. N., Dos Santos, L. M. B., Chouin-Carneiro, T., Pavan, M. G., G.Garcia, G. A., David, M. R., Beier, J. C., Dowell, F. E., Maciel-de-Freitas, R. & Sikulu-Lord, M. T. Rapid, noninvasive detection of Zika virus in Aedes aegypti mosquitoes by near-infrared spectroscopy. *Sci. Adv. *(2018). 10.1126/sciadv.aat0496. 10.1126/sciadv.aat0496 PMC5966221 29806030

[35] Khan RS, Rehman IU (2020). Spectroscopy as a tool for detection and monitoring of Coronavirus (COVID-19). Expert Rev Mol Diagn..

[36] Bertsimas D, Van Parys B (2020). Sparse high-dimensional regression: Exact scalable algorithms and phase transitions. Ann. Stat..

[37] Geinguenaud, F., Militello, V. & Arluison, V. in *Methods in molecular biology (Clifton, N.J.)*, U. Walker, John M.(School of Life and Medical Sciences University of Hertfordshire Hatfield, Hertfordshire, Ed. (2020; 10.1007/978-1-0716-0278-2), vol. 2113, pp. 119–133. 10.1007/978-1-0716-0278-2_10 32006312

[38] Geinguenaud F, Militello V, Arluison V, Arluison V, Wien F (2020). Application of FTIR spectroscopy to analyze RNA structure. RNA spectroscopy methods in molecular biology.

[39] Wood BR (2016). The importance of hydration and DNA conformation in interpreting infrared spectra of cells and tissues. Chem. Soc. Rev..

[40] Banyaya M, Sandbrinkb J, Strombergb R, Graslund A (2004). Characterization of an RNA bulge structure by Fourier transform infrared spectroscopy. Biochem. Biophys. Res. Comm..

[41] Movasaghi Z, Rehman S, Rehman UI (2008). Fourier Transform Infrared (FTIR) spectroscopy of biological tissues. Appl. Spec. Rev..

[42] Dovbeshko GI, Gridina NY, Kruglova EB, Pashchuk OP (2000). FTIR spectroscopy studies of nucleic acid damage. Talanta.

[43] Parker, J., Fowler, N., Walmsley, M. L., Schmidt, T., Charrer, J., Kowaleski, J., Grimes, T., Hoyos, S. &Jack Chen, J. Analytical Sensitivity Comparison between Singleplex Real-Time PCR and a Multiplex PCR Platform for Detecting Respiratory Viruses. *PloS ONE 10*, e0143164, 1–9 (2015). 10.1371/journal.pone.0143164 PMC4646456 26569120

[44] Fung B, Gopez A, Servellita V, Arevalo S, Ho C, Deucher A, Thornborrow E, Chiu C, Miller S (2020). Direct comparison of SARS-CoV- 2 analytical limits of detection across seven molecular assays. J. Clin. Microbiol..

[45] Yu L, Wu S, Hao X, Dong X, Mao L, Pelechano V, Chen W-H, Yin X (2020). Limits of detection of 6 approved RT–PCR kits for the novel SARS-Coronavirus-2 (SARS-CoV-2). Clinical Chem..

[46] Zou L, Ruan F, Huang M (2020). SARS-CoV-2 viral load in upper respiratory specimens of infected patients. N. Engl. J. Med..

[47] Barauna VG, Singh MN, Barbosa LL, Marcarini WD, Vassallo PF, Mill JG, Ribeiro-Rodrigues R, Campos LGG, Warnke PH, Martin FL (2021). ultrarapid on-site detection of SARS-CoV-2 infection using simple ATR-FTIR spectroscopy and an analysis algorithm: High sensitivity and specificity. Anal. Chem..

[48] Nachtigall FM, Pereira A, Trofymchuk OS (2020). Detection of SARS-CoV-2 in nasal swabs using MALDI-MS. Nat. Biotechnol..

