# Supplementary material for: A simple and fast spectroscopy-based technique for Covid-19 diagnosis
Source: Sci Rep. 2021 Aug 18;11:16740. doi: 10.1038/s41598-021-95568-5 (PMC8373901; doi:10.1038/s41598-021-95568-5)
Supplement: Supplementary file 1 — Supplementary Information. [file 41598_2021_95568_MOESM1_ESM.docx]

**Supplementary Materials**

**A simple and fast spectroscopy-based technique for Covid-19 diagnosis**

Driss Lahlou Kitane^1^, Salma Loukman^2^, Nabila Marchoudi^2^, Alvaro Fernandez-Galiana^3^, Fatima Zahra El Ansari^2^, Farah Jouali^2^, Jamal Badir^4^, Jean-Luc Gala^4^, Dimitris Bertsimas^1^, Nawfal Azami^5^, Omar Lakbita^6^, Omar Moudam^6^, Rachid Benhida^6,7*^, Jamal Fekkak^2*^*.*

^1^Operations Research Center, MIT. Muckley Bldg, 1 Amherst St, Cambridge, MA 02142.

^2^Anoual Laboratory, Boulevard d'Alexandrie, 20360 Casablanca, Morocco.

^3^LIGO Laboratory, MIT, 185 Albany Street, Cambridge, MA, 02139.

^4^ Centre for Applied Molecular Technologies (CTMA), Université catholique de Louvain, Louvain, Belgium.

^5^ Photonics Labs, INPT, Madinat Al Irfane, Rabat, Morocco.

^6^ Chemical and Biochemical Sciences department. Mohammed VI Polytechnic University, UM6P. Lot 660,

Hay Moulay Rachid, 43150, Benguerir, Morocco.

^7^ Nice Institute of Chemistry. University Côte d’Azur, Nice, France.

**Table S1.** Primers and probes, real-time RT-PCR for Sars-Cov2 virus

| **Gene** | | **Primer** | **Conditions** |
| --- | --- | --- | --- |
| **E gene** | E_Sarbeco_F | ACAGGTACGTTAATAGTTAATAGCGT | 400 nm per reaction |
|  | E_Sarbeco_P1 | FAM-ACACTAGCCATCCTTACTGCGCTTCG-BBQ | 200 nm per reaction |
|  | E_Sarbeco_R | ATATTGCAGCAGTACGCACACA | 400 nm per reaction |
| **RdRPgene** | RdRp_SARSr-F | GTGARATGGTCATGTGTGGCGG | 600 nM per reaction |
|  | RdRp_SARSr-P1 | FAM-CCAGGTGGWACRTCATCMGGTGATGC-BBQ | 100 nM per reaction and mix with P2 |
|  | RdRP_SARSr-P2 | FAM-CAGGTGGAACCTCATCAGGAGATGC-BBQ | 100 nM per reaction and mix with P1 |
|  | RdRp_SARSr-R | CARATGTTAAASACACTATTAGCATA | 800 nM per reaction |

**Table S2.** FTIR spectroscopy parameters

| Model Name | FT/IR-4600typeA |
| --- | --- |
| Accessory | ATR PRO ONE Accessory |
| Incident angle | 45 deg |
| Light Source Standard Detector | TGS |
| Accumulation | 64 |
| Resolution | 2 cm^-1^ |
| Zero Filling | On |
| Apodization | Cosine |
| Gain | Auto (8) |
| Aperture | Auto (5 mm) |
| Scanning Speed | Auto (2 mm/sec) |
| Filter | Auto (30000 Hz) |
| Data array type | Linear data array |
| Horizontal axis | Wavenumber [cm^-1^] |
| Vertical axis | Absorbance |
| Start | 399.675 cm^-1^ |
| End | 8000.25 cm^-1^ |
| Data interval | 0.482117 cm^-1^ |
| Data points | 15766 |


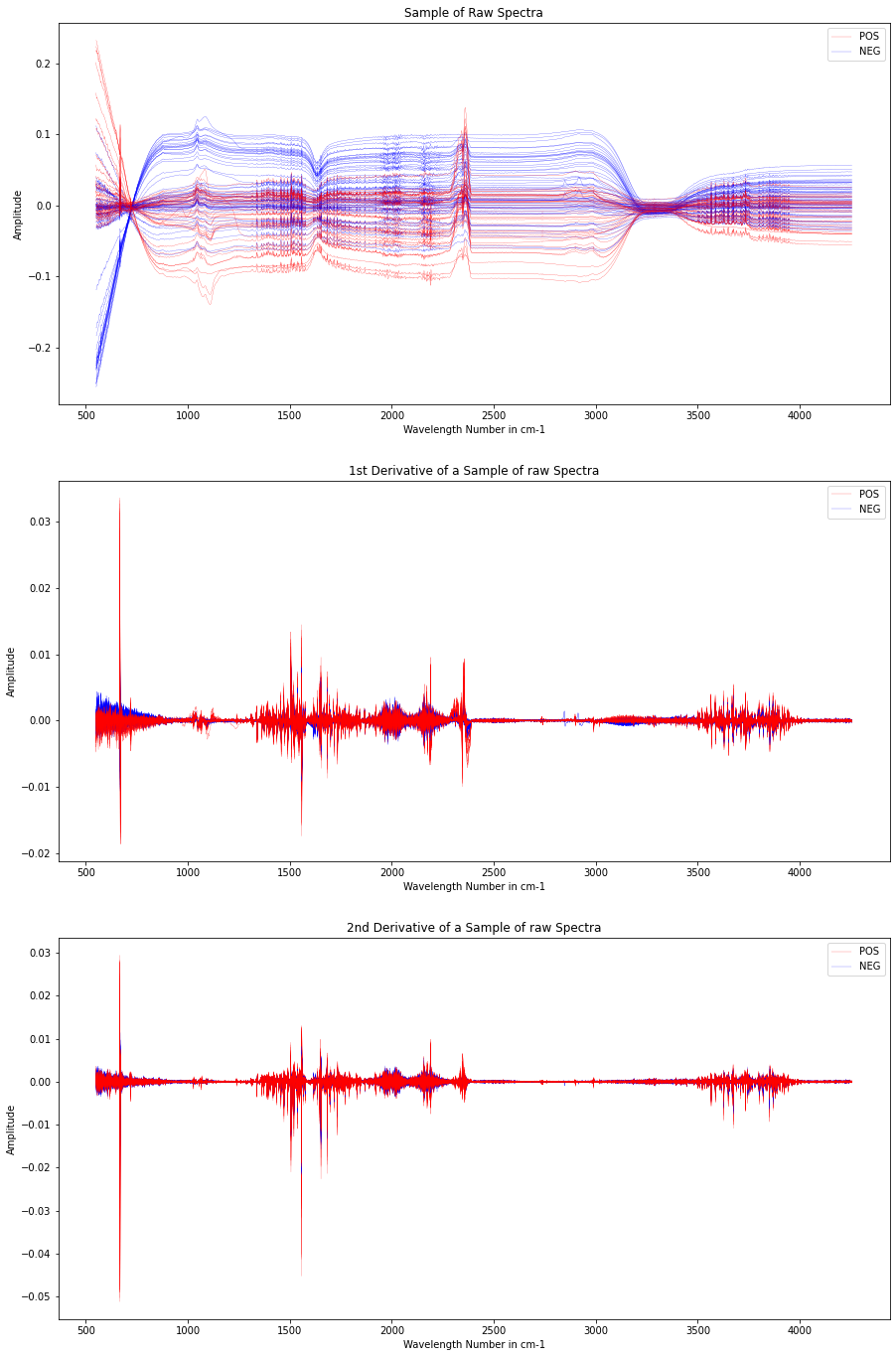


**Fig. S1**. Raw Spectra


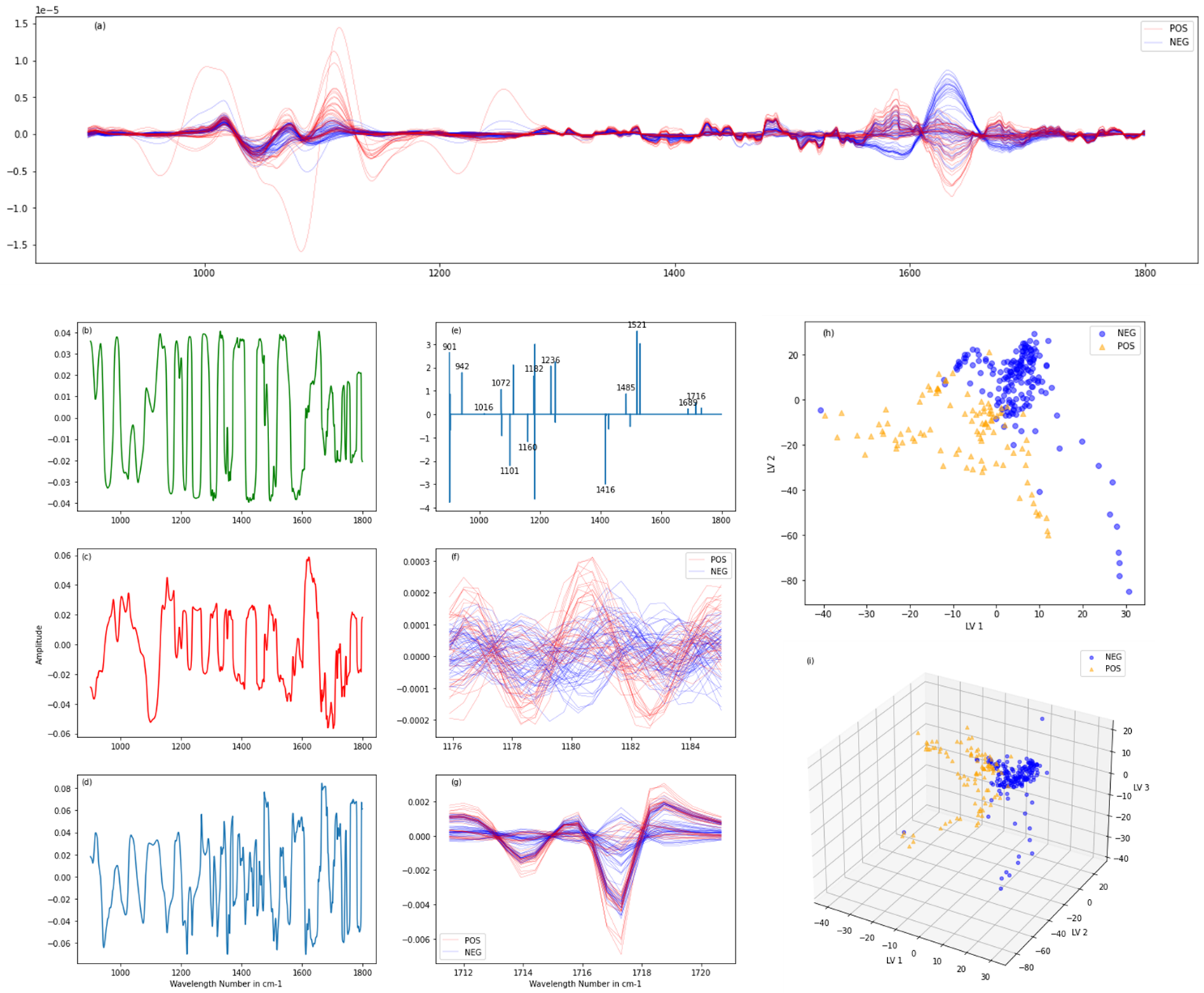


**Fig. S2.** Detection of SARS-CoV-2 with multivariable analyss. (**a)**-Sample of 2^nd^ derivative (1800-900cm^-1^) of Savitzky-Gola smoothened positive and negative spectra. (**b,c and d**) - First 3 latent variables of PLS-DA **(e)** Coefficients of variables selected by sparse classification algorithm of second derivative of raw spectra **(f,g)** Zooms on regions indicated by sparse classification. **(h)**-Projection of the 280 spectra used according to the first 2 latent variables obtained using PLS regression. **(i)** Projection of the 280 spectra used according to the first 3 latent variables obtained using PLS regression.


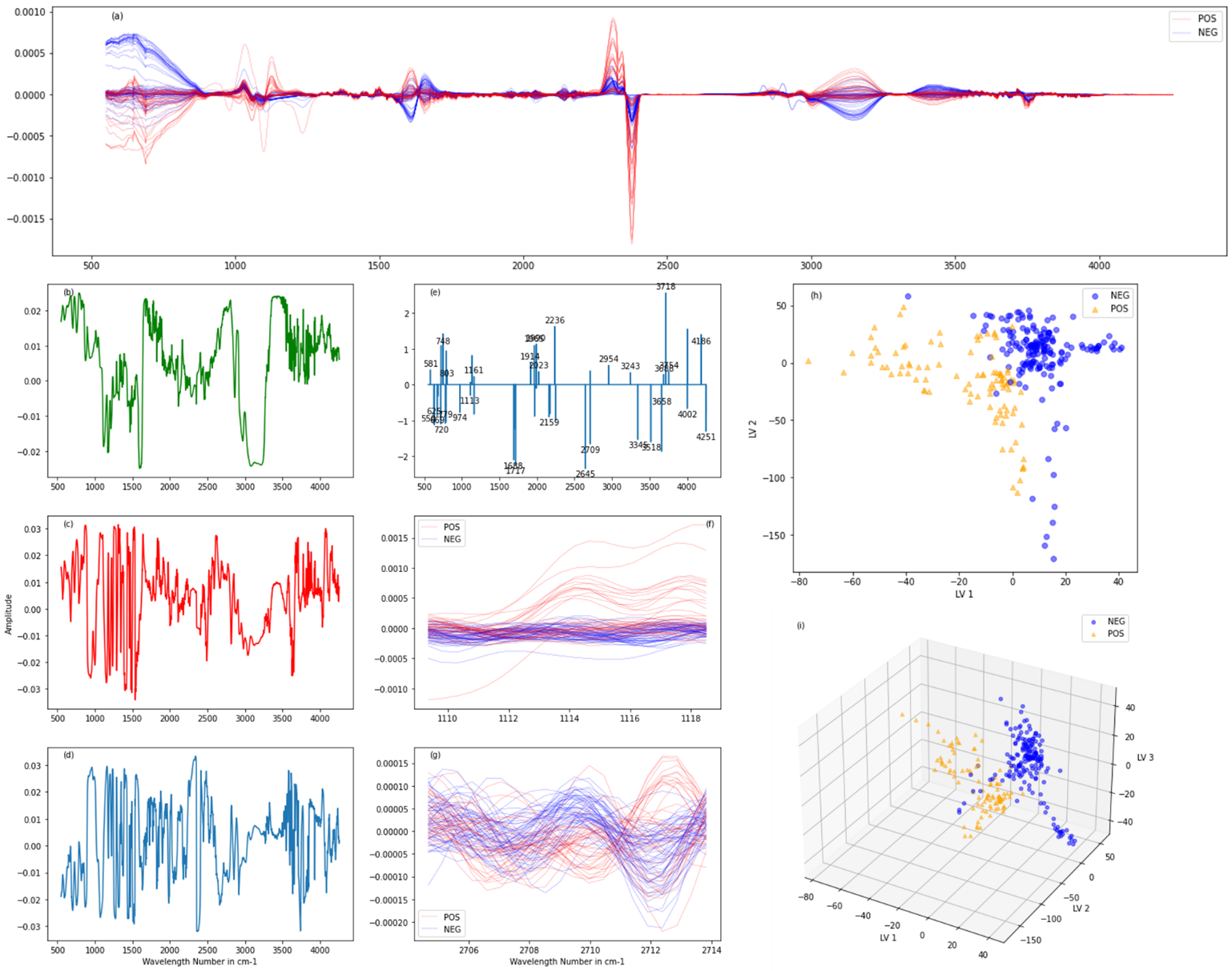


**Fig. S3.** Detection of SARS-CoV-2 with multivariable analysis. (**a)**-Sample of 1^st^ derivative (4500-550cm^-1^) of Savitzky-Gola smoothened positive and negative spectra. (**b, c and d**) - First 3 latent variables of PLS-DA **(e)**Coefficients of variables selected by sparse classification algorithm of first derivative of raw spectra **(f,g)** Zooms on regions indicated by sparse classification. **(h)**-Projection of the 280 spectra used according to the first 2 latent variables obtained using PLS regression. **(i)** Projection of the 280 spectra used according to the first 3 latent variables obtained using PLS regression.


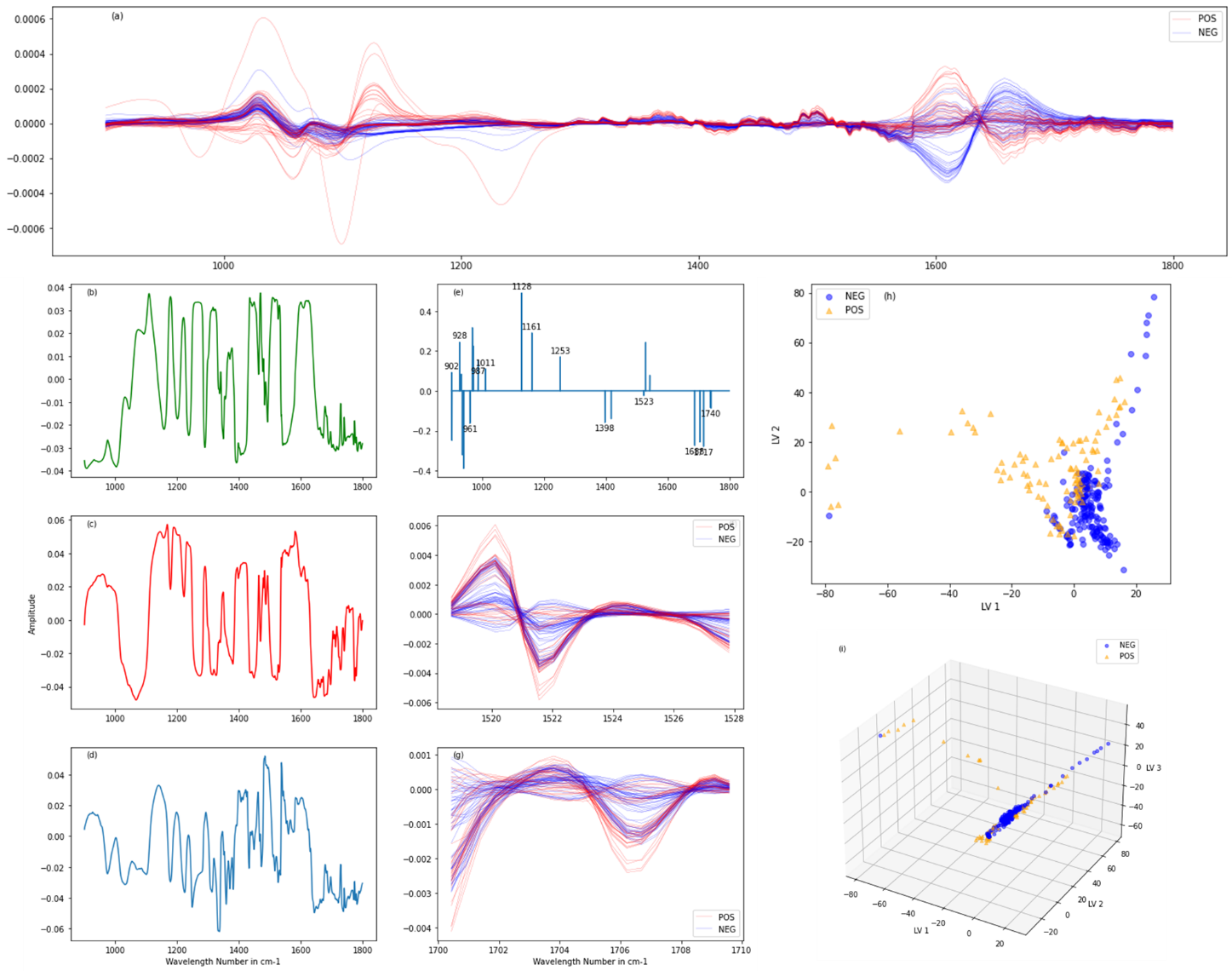


**Fig. S4.** Detection of SARS-CoV-2 with multivariable analysis. (**a)**-Sample of 1^st^ derivative (**1800-900cm^-1^**) of Savitzky-Gola smoothened positive and negative spectra. (**b,c and d**) - First 3 latent variables of PLS-DA **(e)**Coefficients of variables selected by sparse classification algorithm of first derivative of raw spectra **(f,g)** Zooms on regions indicated by sparse classification. **(h)**-Projection of the 280 spectra used according to the first 2 latent variables obtained using PLS regression. **(i)** Projection of the 280 spectra used according to the first 3 latent variables obtained using PLS regression.

**Benchmark of Predictive Power of Selected Algorithms on the 1800-900 cm^-1^region**

We randomly draw 185 samples out of the 280 available for training and 95 for testing. We randomly draw the training set 25 times and report the results below.

**Raw Spectra**

**Baseline Corrected Spectra**

**1^st^ Derivative of Spectra**


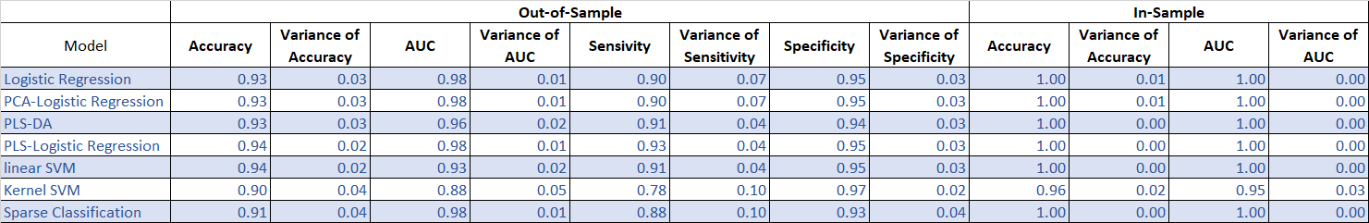


**2^nd^ Derivative of Spectra**


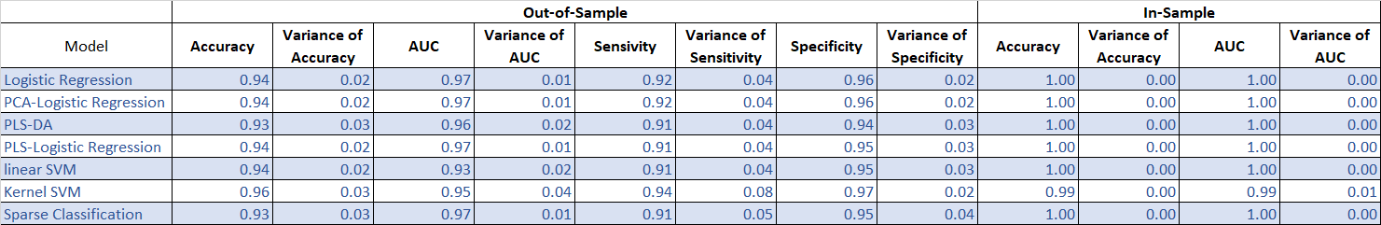


**Benchmark of Predictive Power of Selected Algorithms on the 4500-600 cm^-1^ region**

We randomly draw 185 samples out of the 280 available for training and 95 for testing. We randomly draw the training set 25 times and report the results below.

**Raw Spectra**


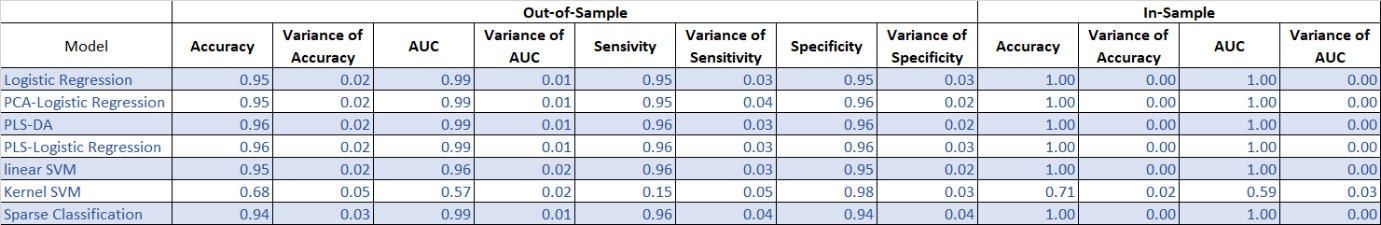


**Baseline Corrected Spectra**


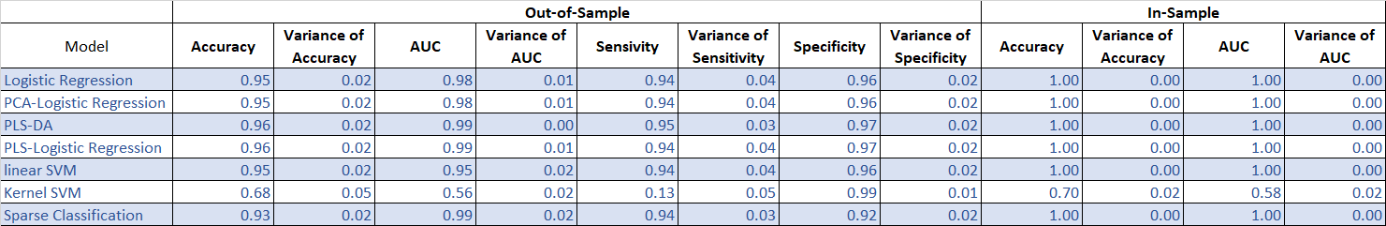


**1^st^ Derivative of Spectra**


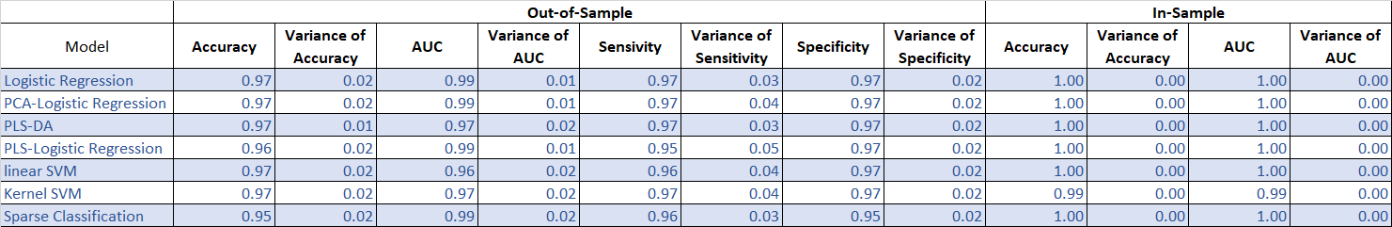


**2^nd^ Derivative of Spectra**


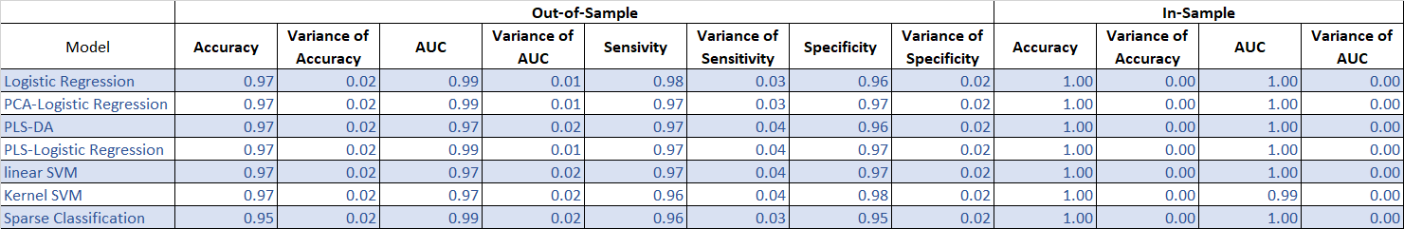


The quality of extracted RNA is measured with Biochrom-Nanovue equipment. Table S3 shows a set of fresh samples, just after RNA extraction, while in Figure 3 is reported the quality of RNAs after several days.

**Table S3.** Concentration and quality of freshly extracted RNAs used in RT-PCR and FT-IR

**
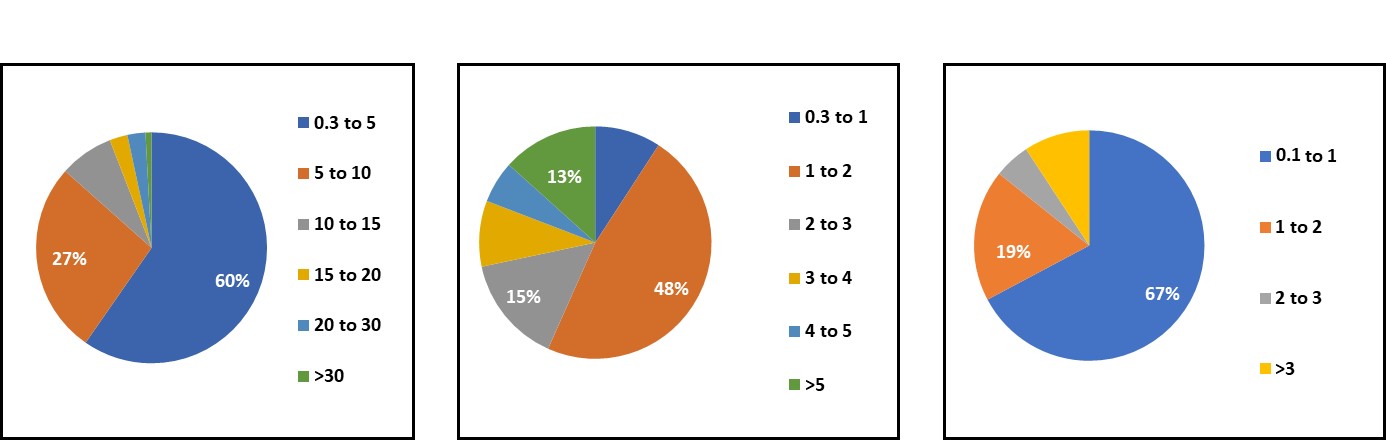
**

**Fig. S5.** (a) Distribution of the RNA concentration over the samples after several days at -20°C. (b) A260/A280 extinction ratio distribution (c) A260/A230 extinction ratio distribution
